# Supplementary material for: A set of multi-entry identification keys to African frugivorous flies (Diptera, Tephritidae)
Source: Zookeys. 2014 Jul 24;(428):97–108. doi: 10.3897/zookeys.428.7366 (PMC4143993; doi:10.3897/zookeys.428.7366)
Supplement: Supplementary material 4 — Key to Capparimyia [file zookeys-428-097-s004.zip › SF4_ZooKeys_key to Capparimyia/lucid_player/help/help/feature_gallery_view.htm]

Feature Gallery View


# Feature Gallery View

The feature gallery view provides an alternative view of multistate
features. Instead of a series of states being displayed in the tree below
a multistate feature, the feature gallery displays a tabular view of thumbnails
representing the states of the feature.

Clicking on a thumbnail in the gallery will select it�s related state,
the icons overlaid on the thumbnails can be used to open any html or image
files attached to the state.

To enable the gallery view, select the *Image
Gallery* option under the *Display*
submenu located within the *Features*
menu.
